# Circular RNA (circRNA) in Alzheimer's disease (AD)

**DOI:** 10.3389/fgene.2013.00307

**Published:** 2013-12-31

**Authors:** Walter J. Lukiw

**Affiliations:** LSU Neuroscience Center and Departments of Neurology and Ophthalmology, Louisiana State University Health Science CenterNew Orleans, LA, USA

**Keywords:** micro RNA, miRNA-7, circular RNAs, evolution, gene regulation, Alzheimer's disease, transcriptome, hippocampal CA1

**Figure 1:**
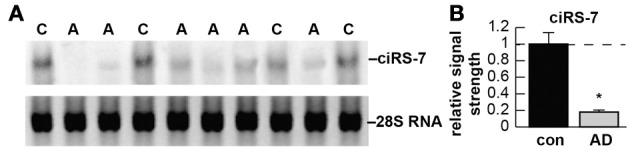
**(A)** Detection of circRNA for miRNA-7 (ciRS-7) in sporadic Alzheimer's disease (AD) and age-matched control hippocampal CA1 [control **(C)** *N* = 4; AD **(A)** *N* = 6]; the single upper ciRS-7 (~1400 nt) band contains ~70 selectively conserved miRNA-7 binding sites as previously described (Hansen et al., 2013); a lower 28S RNA served as an internal loading control; all samples depleted of rRNA were treated with 50 units of RNaseR prior to electrophoresis RNaseR is a processive, Mg++-dependent hydrolytic exoribonuclease that degrades linear but not circular RNA; see Hansen et al. (2013); predicted circular transcripts consistently resisted an RNaseR challenge; 30 ug total AD and control hippocampal CA1 RNA was separated on agarose gels, transferred and probed with biotinylated or radiolabeled miRNA-7 probes as previously described (Colangelo et al., 2002; Hansen et al., 2013); detection was performed using a nonisotopic BrightStar BioDetect Kit (Ambion, Austin, TX; detection limit ~100 fg) or by standard autoradiography (Lukiw et al., 1992); **(B)** AD ciRS-7 is significantly reduced to about 0.18-fold of control (*con*) in a study of 20 control and AD (*AD*) hippocampal CA1 samples; this implicates loss of miRNA-7 sponge effects, an ambient up-regulation of miRNA-7 (as is observed), and down-regulation of a family of miRNA-7-sensitive mRNAs in the sporadic AD brain; other circRNAs may be involved; there were no significant differences between age for control or AD tissues [mean ± 1 standard deviation *(SD)* = 75.4 ± 8.3 year vs. 77.5 ± 7.6 year]; all AD cases were for moderate-to-advanced stages of AD; all post-mortem intervals were 2.1 h or less (Colangelo et al., 2002); there were no significant differences in age, ApoE allele status, RNA quality (all RIN values were 8.1–9.0) or yield between the control or AD groups (*p* > 0.05, ANOVA); a dashed horizontal line at 1.0 indicates homeostatic (control) ciRS-7 levels for ease of comparison; ^*^p < 0.001 (ANOVA).

Circular RNAs (circRNAs) are a naturally occurring family of noncoding RNAs (ncRNAs) highly represented in the eukaryotic transcriptome. Recently characterized, traditional methods of RNA detection and analysis requiring a free 5′ or 3′ ribonucleotide terminus may have significantly underestimated circRNA abundance and significance in eukaryotic *cells* (Salzman et al., 2012; Wilusz and Sharp, 2013; unpublished observations). Intrinsically resistant to exonucleolytic RNA decay, circRNAs appear to be enriched in mammalian brain tissues (Hansen et al., 2013; Memczak et al., 2013). Interestingly, specific ncRNAs such as the evolutionary ancient microRNA-7 (miRNA-7; chr 9q21.32; an important post-transcriptional regulator of human brain gene expression), are not only highly abundant in human brain, but are also associated with a circRNA for miRNA-7 (ciRS-7), in the same tissues; ciRS-7 contains multiple, tandem anti-miRNA-7 sequences (Burmistrova et al., 2007; Hansen et al., 2013; Lukiw et al., 2013). ciRS-7 thereby acts as a kind of endogenous, competing, anti-complementary miRNA “sponge” to adsorb, and hence quench, normal miRNA-7 functions. Using Northern blot hybridization techniques and the circularity-sensitive circRNA probe RNaseR we here provide initial evidence of a mis-regulated miRNA-7-circRNA system in the sporadic Alzheimer's disease (AD) hippocampal CA1 region (Figure 1). Deficits in ciRS-7, and ciRS-7 “sponging activities” might be expected to increase ambient miRNA-7 levels in AD-affected brain cells, as is observed, to ultimately contribute to the down-regulation of selective miRNA-7-sensitive messenger RNA (mRNA) targets (Cogswell et al., 2008; unpublished observations). The presence of up-regulated miRNA-7, due to a deficiency in ciRS-7 “sponging” effects, has high probability to down-regulate AD-relevant targets, such as, for example, the ubiquitin protein ligase A (UBE2A; miRNA-7-UBE2A mRNA energy of association, *E*_*A*_ = −22.86 kcal/mol). UBE2A, an autophagic, phagocytic protein essential in the clearance of amyloid peptides in AD and other progressive inflammatory degenerations of the human CNS, is depleted in AD brain (Bingol and Sheng, 2011; Lonskaya et al., 2013). Such miRNA-mRNA regulatory systems mediated by a family of cell- and/or tissue-enriched circRNAs may represent another important layer of epigenetic control over gene expression in health and disease. Indeed, technological advancement and recent discoveries in the field of ncRNAs continue to challenge our basic doctrines of nucleic acid biochemistry and evolutionary biology. Deficits in other circRNA-mediated “miRNA sponging systems” and ambient up-regulation of specific inducible miRNAs may help explain the widely observed, generalized and progressive down-regulation of gene expression that is characteristic of the sporadic AD brain (Loring et al., 2001; Colangelo et al., 2002; Ginsberg et al., 2012; Lukiw, 2013).
